# P75 - The role of the pediatric research nurse in ensuring successful recruitment and retention of research participants for the UBIOPRED pediatric asthma study

**DOI:** 10.1186/2045-7022-4-S1-P130

**Published:** 2014-02-28

**Authors:** Jane Martin, Ruth Morris, Zaraquiza Zolkipli, Graham Roberts

**Affiliations:** 1 University Hospital Southampton NHS Foundation Trust, University of Southampton, Southampton, United Kingdom

## Introduction

The UBIOPRED study (Unbiased BIOmarkers for the Prediction of REspiratory Diseases) is a longitudinal asthma research study. It will identify phenotype/’omic handprints to improve our understanding of severe asthma and identify potential targets for new pharmacotherapies.

The aim of this poster is to illustrate how the research team successfully recruited and retained the Southampton portion of the paediatric UBIOPRED cohort using an acronymic approach.

## Methods

A simple acronym was developed by the NIHR Southampton Wellcome Trust Clinical Research Facility (WTCRF) nurses to enable successful recruitment and retention of participants:

• **A**pproach –Participants were usually approached during their outpatients’ appointment. This ensured neutral ground, so the study could be discussed with their consultant.

• **B**elief – The research nurses believed in UBIOPRED, which instilled confidence when discussing the study with potential recruits.

• **C**ommitment – Retention was only possible through the commitment of participants and nurses. Study visits were coincided with clinic appointments whenever possible to reduce inconvenience.

• **D**edication – The research nurses went to great lengths to ensure the participants’ needs were met.

• **E**xperience – The participants’ research experience needed to be positive to ensure retention. Participants were supported if they had anxieties and were never pressurised to undertake research procedures.

• **F**un –Age appropriate fun was key to each study appointment.

## Results

**Figure 1:**
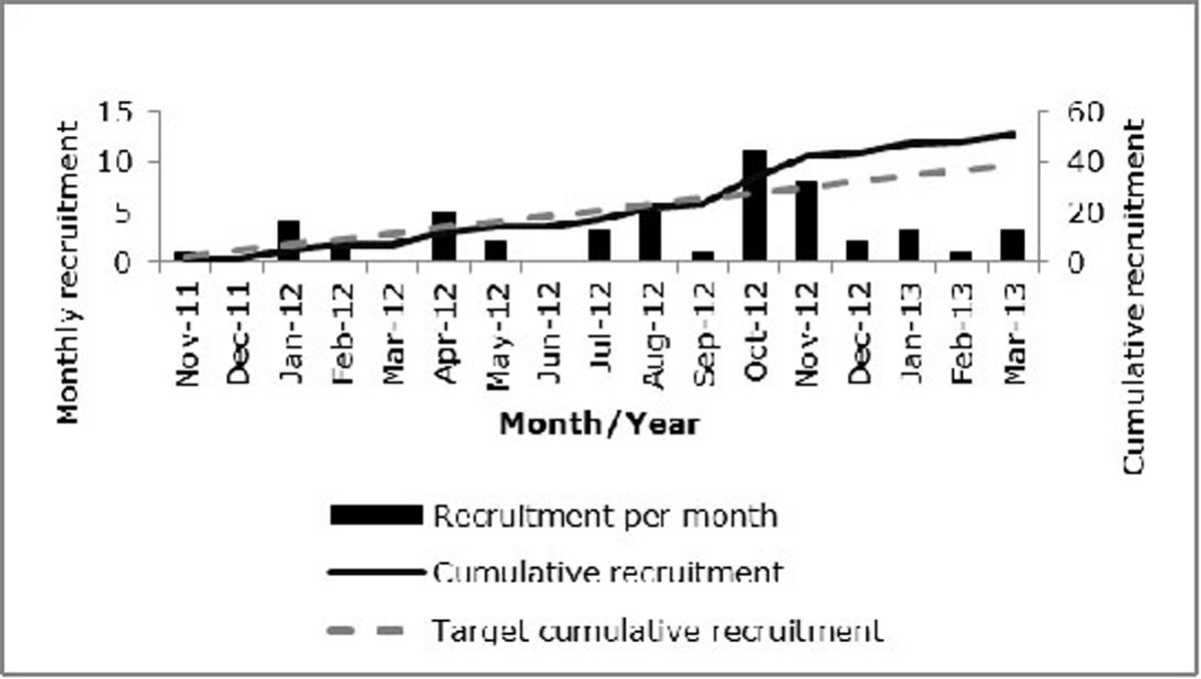
UBIOPRED paediatric recruitment

**Figure 2:**
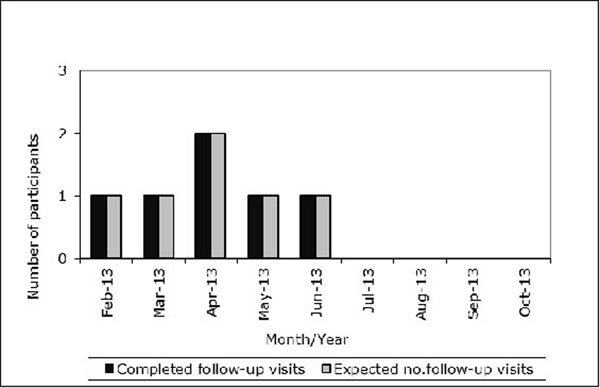
Completed/expected longitudinal visits

The final recruitment figures for the UBIOPRED paediatric cohort at Southampton exceeded the target set (figure 1). At present, all participants have returned for longitudinal visits within the expected time frame (figure 2). The feedback from the parents and participants has demonstrated that the research has impacted positively on their lives often resulting in better education of their asthma and improved disease control.

## Conclusions

Using an ABCDEF approach, the children’s asthma and allergy research nurses have exceeded their recruitment target and successfully followed up participants on the UBIOPRED study.

